# 3,5-Dichloro-2-hydroxy­benzaldehyde

**DOI:** 10.1107/S1600536808013901

**Published:** 2008-05-14

**Authors:** Ying Fan, Wei You, Jian-Lan Liu, Hui-Fen Qian, Wei Huang

**Affiliations:** a College of Sciences, Nanjing University of Technology, Nanjing 210009, People’s Republic of China; b State Key Laboratory of Coordination Chemistry, Coordination Chemistry Institute, School of Chemistry and Chemical Engineering, Nanjing University, Nanjing 210093, People’s Republic of China

## Abstract

The title compound, C_7_H_4_Cl_2_O_2_, exhibits a layer crystal structure; mol­ecules within each layer are linked by weak C—H⋯O inter­molecular hydrogen bonds. There is also an intramolecular O—H⋯O hydrogen bond.

## Related literature

For a related compound, see: Fan *et al.* (2008 ▶).
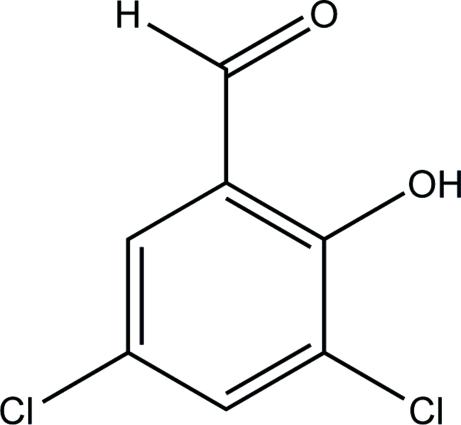


## Experimental

### 

#### Crystal data

C_7_H_4_Cl_2_O_2_
*M* *_r_* = 191.00
Monoclinic, 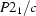

*a* = 8.3359 (16) Å
*b* = 13.884 (3) Å
*c* = 7.2341 (14) Å
β = 114.519 (2)°
*V* = 761.7 (3) Å^3^
*Z* = 4
Mo *K*α radiation
μ = 0.79 mm^−1^
*T* = 291 (2) K
0.14 × 0.12 × 0.10 mm

#### Data collection

Bruker SMART CCD area-detector diffractometer
Absorption correction: multi-scan (*SADABS*; Bruker, 2000 ▶) *T*_min_ = 0.897, *T*_max_ = 0.925
4063 measured reflections
1487 independent reflections
1181 reflections with *I* > 2σ(*I*)
*R*_int_ = 0.055

#### Refinement

*R*[*F* ^2^ > 2σ(*F* ^2^)] = 0.036
*wR*(*F* ^2^) = 0.097
*S* = 0.99
1487 reflections
101 parameters
H-atom parameters constrained
Δρ_max_ = 0.27 e Å^−3^
Δρ_min_ = −0.23 e Å^−3^

### 

Data collection: *SMART* (Bruker, 2000 ▶); cell refinement: *SAINT* (Bruker, 2000 ▶); data reduction: *SAINT*; program(s) used to solve structure: *SHELXTL* (Sheldrick, 2008 ▶); program(s) used to refine structure: *SHELXTL*; molecular graphics: *SHELXTL*; software used to prepare material for publication: *SHELXTL*.

## Supplementary Material

Crystal structure: contains datablocks global, I. DOI: 10.1107/S1600536808013901/at2566sup1.cif

Structure factors: contains datablocks I. DOI: 10.1107/S1600536808013901/at2566Isup2.hkl

Additional supplementary materials:  crystallographic information; 3D view; checkCIF report

## Figures and Tables

**Table 1:** Hydrogen-bond geometry (Å, °)

*D*—H⋯*A*	*D*—H	H⋯*A*	*D*⋯*A*	*D*—H⋯*A*
O2—H2⋯O1	0.82	1.92	2.630 (2)	145
C4—H4⋯O1^i^	0.93	2.51	3.428 (3)	168
C6—H6⋯O2^ii^	0.93	2.56	3.394 (3)	149

Symmetry codes: (i) 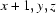; (ii) 
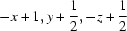
.

**Fig. 1.**
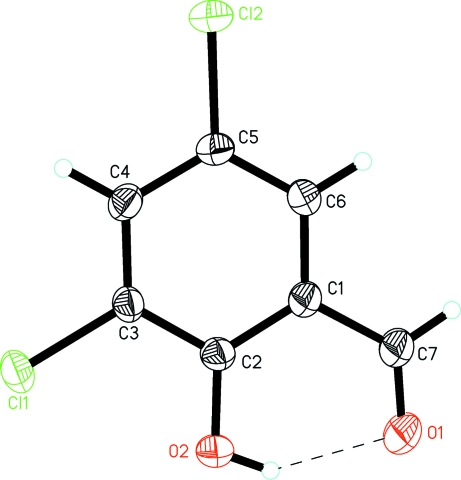
An ORTEP drawing of the title compound with the atom-numbering scheme. Displacement ellipsoids are drawn at the 30% probability level.

**Fig. 2.**
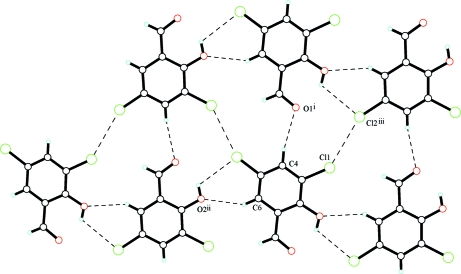
A perspective view of the intralayer intermolecular hydrogen-bond contacts among molecules in the title compound. Hydrogen bonds and Cl···Cl interactions are shown as dashed lines. [Symmetry codes: (i) x+1, y, z; (ii) -x + 1, y+1/2, -z+1/2; (iii) -x+2, y - 1/2, -z + 1/2].

**Fig. 3.**
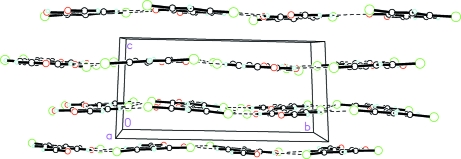
A perspective view of the layer packing structure of (I) together with the unit cell.

## supplementary crystallographic information

### Comment

We have newly reported the X-ray single-crystal structure of 3,5-dibromo-2-hydroxybenzaldehyde (Fan *et al.*, 2008). In this paper, we report the X-ray single-crystal structure of 3,5-dichloro-2- hydroxybenzaldehyde.

The molecular structure of (I) is illustrated in Fig. 1. The selected bond distances and bond angles are normal. Different from 3,5-dibromo-2-hydroxybenzaldehyde, there is only one crystallographically independent molecule in the asymmetric unit. The molecular geometry of slicylaldehyde unit of (I) is comparable with that of 3,5-dibromo-2-hydroxybenzaldehyde.

In the crystal packing of (I), there are two sets of molecules with the dihedral angle of 6.52 (2) ° and molecules in every layer are linked by intermolecular CO—H···O hydrogen bondings (Fig. 2). A layer packing structure is formed with the mean interlayer separation of 3.428 (2) Å (Fig. 3.). However, no π–π stacking interactions can be observed in (I), which is different from those in 3,5-dibromo-2-hydroxybenzaldehyde.

### Experimental

The title compound was obtained as received. Single crystals suitable for X-ray diffraction measurement were formed after 6 days in methanol by slow evaporation at room temperature in air. Analysis calculated for C_7_H_4_O_2_Cl_2_: C 44.02, H 2.11%. Found: C 44.18, H, 2.24%. FT—IR (KBr pellets, cm^-1^): 3066(*versus*), 2856(*s*), 1666(*versus*), 1604(*m*), 1428(*s*), 1375(*versus*), 1276(*s*), 1208(*s*), 1171(*s*), 1103(*m*), 935(*s*), 891(*versus*), 735(*s*), 703(*s*), 566(*m*), and 515(*m*).

### Refinement

The H atoms were placed in geometrically idealized positions (C—H = 0.93 Å and O—H = 0.82 Å) and refined as riding atoms, with *U*_iso_(H) = 1.2*U*_eq_(C) and *U*_iso_(H) = 1.5*U*_eq_(O).

### Crystal data

**Table:**

C_7_H_4_Cl_2_O_2_	*F*_000_ = 384
*M**_r_* = 191.00	*D*_x_ = 1.666 Mg m^−^^3^
Monoclinic, *P*2_1_/*c*	Mo *K*α radiation λ = 0.71073 Å
Hall symbol: -P 2ybc	Cell parameters from 1691 reflections
*a* = 8.3359 (16) Å	θ = 2.7–26.8º
*b* = 13.884 (3) Å	µ = 0.79 mm^−^^1^
*c* = 7.2341 (14) Å	*T* = 291 (2) K
β = 114.519 (2)º	Block, yellow
*V* = 761.7 (3) Å^3^	0.14 × 0.12 × 0.10 mm
*Z* = 4	

### Data collection

**Table:**

Bruker SMART CCD area-detector diffractometer	1487 independent reflections
Radiation source: fine-focus sealed tube	1181 reflections with *I* > 2σ(*I*)
Monochromator: graphite	*R*_int_ = 0.055
*T* = 291(2) K	θ_max_ = 26.0º
φ and ω scans	θ_min_ = 2.7º
Absorption correction: multi-scan(SADABS; Bruker, 2000)	*h* = −10→10
*T*_min_ = 0.898, *T*_max_ = 0.925	*k* = −16→16
4063 measured reflections	*l* = −7→8

### Refinement

**Table:**

Refinement on *F*^2^	Secondary atom site location: difference Fourier map
Least-squares matrix: full	Hydrogen site location: inferred from neighbouring sites
*R*[*F*^2^ > 2σ(*F*^2^)] = 0.036	H-atom parameters constrained
*wR*(*F*^2^) = 0.097	*w* = 1/[σ^2^(*F*_o_^2^) + (0.0536*P*)^2^] where *P* = (*F*_o_^2^ + 2*F*_c_^2^)/3
*S* = 0.99	(Δ/σ)_max_ < 0.001
1487 reflections	Δρ_max_ = 0.27 e Å^−^^3^
101 parameters	Δρ_min_ = −0.23 e Å^−^^3^
Primary atom site location: structure-invariant direct methods	Extinction correction: none

### Special details

**Table:**

Experimental. The structure was solved by direct methods (Bruker, 2000) and successive difference Fourier syntheses.
Geometry. All e.s.d.'s (except the e.s.d. in the dihedral angle between two l.s. planes) are estimated using the full covariance matrix. The cell e.s.d.'s are taken into account individually in the estimation of e.s.d.'s in distances, angles and torsion angles; correlations between e.s.d.'s in cell parameters are only used when they are defined by crystal symmetry. An approximate (isotropic) treatment of cell e.s.d.'s is used for estimating e.s.d.'s involving l.s. planes.
Refinement. Refinement of *F*^2^ against ALL reflections. The weighted *R*-factor *wR* and goodness of fit *S* are based on *F*^2^, conventional *R*-factors *R* are based on *F*, with *F* set to zero for negative *F*^2^. The threshold expression of *F*^2^ > σ(*F*^2^) is used only for calculating *R*-factors(gt) *etc*. and is not relevant to the choice of reflections for refinement. *R*-factors based on *F*^2^ are statistically about twice as large as those based on *F*, and *R*- factors based on ALL data will be even larger.

### Fractional atomic coordinates and isotropic or equivalent isotropic displacement parameters (Å^2^)

**Table:**

	*x*	*y*	*z*	*U*_iso_*/*U*_eq_	
C1	0.4748 (2)	0.35190 (14)	0.2605 (3)	0.0419 (5)	
C2	0.5509 (2)	0.26110 (14)	0.2721 (3)	0.0383 (4)	
C3	0.7213 (2)	0.25643 (14)	0.2822 (3)	0.0379 (4)	
C4	0.8119 (2)	0.33830 (13)	0.2774 (3)	0.0422 (5)	
H4	0.9261	0.3341	0.2858	0.051*	
C5	0.7313 (2)	0.42742 (15)	0.2599 (3)	0.0422 (5)	
C6	0.5658 (3)	0.43474 (15)	0.2547 (3)	0.0445 (5)	
H6	0.5145	0.4949	0.2474	0.053*	
C7	0.2987 (3)	0.35981 (17)	0.2606 (3)	0.0536 (6)	
H7	0.2525	0.4210	0.2579	0.064*	
Cl1	0.81885 (7)	0.14449 (4)	0.29976 (9)	0.0542 (2)	
Cl2	0.84604 (7)	0.52989 (4)	0.24617 (10)	0.0612 (2)	
O1	0.21042 (19)	0.29062 (13)	0.2640 (3)	0.0678 (5)	
O2	0.46796 (18)	0.17843 (10)	0.2737 (2)	0.0519 (4)	
H2	0.3694	0.1904	0.2670	0.078*	

### Atomic displacement parameters (Å^2^)

**Table:**

	*U*^11^	*U*^22^	*U*^33^	*U*^12^	*U*^13^	*U*^23^
C1	0.0364 (10)	0.0424 (12)	0.0505 (11)	0.0021 (8)	0.0216 (9)	0.0031 (9)
C2	0.0405 (10)	0.0354 (11)	0.0423 (10)	−0.0011 (8)	0.0206 (8)	0.0018 (8)
C3	0.0389 (10)	0.0360 (11)	0.0428 (10)	0.0048 (8)	0.0209 (8)	0.0000 (8)
C4	0.0357 (10)	0.0470 (14)	0.0483 (12)	0.0007 (8)	0.0218 (9)	0.0033 (9)
C5	0.0410 (11)	0.0383 (11)	0.0492 (11)	−0.0052 (8)	0.0209 (9)	0.0034 (9)
C6	0.0432 (11)	0.0367 (11)	0.0568 (12)	0.0050 (8)	0.0240 (10)	0.0045 (9)
C7	0.0427 (12)	0.0502 (14)	0.0746 (15)	0.0042 (10)	0.0310 (11)	0.0076 (11)
Cl1	0.0576 (4)	0.0406 (3)	0.0719 (4)	0.0126 (2)	0.0344 (3)	0.0019 (2)
Cl2	0.0515 (3)	0.0438 (4)	0.0914 (5)	−0.0092 (2)	0.0327 (3)	0.0102 (3)
O1	0.0463 (8)	0.0642 (12)	0.1052 (13)	−0.0007 (8)	0.0437 (8)	0.0073 (9)
O2	0.0475 (8)	0.0370 (8)	0.0773 (10)	−0.0056 (6)	0.0319 (8)	0.0020 (7)

### Geometric parameters (Å, °)

**Table:**

C1—C6	1.388 (3)	C4—H4	0.9300
C1—C2	1.398 (3)	C5—C6	1.369 (3)
C1—C7	1.472 (3)	C5—Cl2	1.740 (2)
C2—O2	1.342 (2)	C6—H6	0.9300
C2—C3	1.393 (2)	C7—O1	1.217 (3)
C3—C4	1.373 (3)	C7—H7	0.9300
C3—Cl1	1.7340 (19)	O2—H2	0.8200
C4—C5	1.388 (3)		
			
C6—C1—C2	120.59 (18)	C5—C4—H4	120.3
C6—C1—C7	119.71 (18)	C6—C5—C4	120.85 (18)
C2—C1—C7	119.68 (18)	C6—C5—Cl2	120.63 (16)
O2—C2—C3	118.51 (17)	C4—C5—Cl2	118.52 (14)
O2—C2—C1	123.29 (16)	C5—C6—C1	119.68 (18)
C3—C2—C1	118.20 (17)	C5—C6—H6	120.2
C4—C3—C2	121.30 (17)	C1—C6—H6	120.2
C4—C3—Cl1	119.86 (13)	O1—C7—C1	123.6 (2)
C2—C3—Cl1	118.84 (15)	O1—C7—H7	118.2
C3—C4—C5	119.34 (17)	C1—C7—H7	118.2
C3—C4—H4	120.3	C2—O2—H2	109.5

### Hydrogen-bond geometry (Å, °)

**Table:**

*D*—H···*A*	*D*—H	H···*A*	*D*···*A*	*D*—H···*A*
O2—H2···O1	0.82	1.92	2.630 (2)	145
C4—H4···O1^i^	0.93	2.51	3.428 (3)	168
C6—H6···O2^ii^	0.93	2.56	3.394 (3)	149

Symmetry codes: (i) *x*+1, *y*, *z*; (ii) −*x*+1, *y*+1/2, −*z*+1/2.
